# Complete Heart Block Causing Takotsubo Syndrome: A Case Report

**DOI:** 10.7759/cureus.24759

**Published:** 2022-05-05

**Authors:** Jaswanth R Jasti, Philip Petrasko, Vernonica A Stys, Mark F Petrasko, Muhammad Hamza Saad Shaukat, Adam Stys

**Affiliations:** 1 Internal Medicine, University of South Dakota Sanford School of Medicine, Sioux Falls, USA; 2 Cardiology, University of South Dakota Sanford School of Medicine, Sioux Falls, USA; 3 Cardiology, University of South Dakota, Sanford Heart Hospital, Sioux Falls, USA

**Keywords:** apical ballooning syndrome, broken heart synrome, catecholamine, pacemaker, apical akinesia, takotsubo syndrome, complete heart block

## Abstract

The association of complete atrioventricular (AV) block with Takotsubo syndrome (TTS) is well known, but the cause-and-effect relationship has not been determined. We present the case of a 91-year-old female with complete AV block who went untreated for over a year and later developed Takotsubo syndrome. Reversal of wall movement defects was seen after a permanent pacemaker was implanted, and routine follow-up showed that the implanted pacemaker worked normally.

## Introduction

Takotsubo syndrome (TTS) is a reversible form of acute left ventricular systolic dysfunction that most commonly affects postmenopausal women. TTS is frequently preceded by either physical or emotional stress and manifests with electrocardiogram (EKG) findings such as ST-segment elevations and T-wave inversions and less frequently with chest pain and arrhythmias but without angiographic evidence of obstructive coronary artery disease or acute plaque rupture [1,2]. The exact cause is unknown, but catecholamine excess and sympathetic nervous system overactivity are potential contributors [2,3]. Several cases of TTS associated with complete heart block (CHB) have been reported in the literature [4-8]. Their possible cause-and-effect relationship, however, has yet to be explained. Our case study looks at a patient who had CHB and developed TTS after going untreated.

## Case presentation

**Figure 1:**
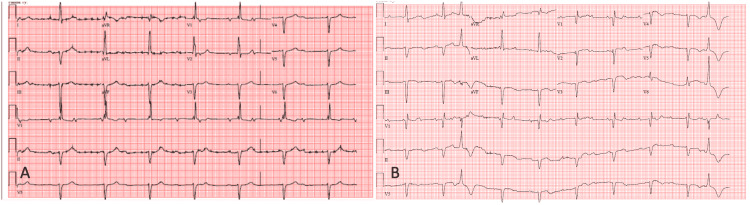
(A, B) Both show complete AV dissociation. There is a relatively narrow QRS with intermittent ventricular ectopy.

A 91-year-old woman with a history of heart failure with preserved ejection fraction (HFpEF), untreated CHB, hypertension, hyperlipidemia, and osteoarthritis presented to the emergency department with a history of a fall and complaints of dyspnea on exertion and fatigue for three weeks in the last week of May 2021. Vitals on presentation showed a heart rate of 55 bpm, blood pressure of 152/76 mm Hg, and arterial oxygen saturation of 95% on room air. Past surgical, social, and family history were noncontributory. She was not on any rate-controlling drug, and her home medications included enalapril, furosemide, and Tylenol as needed for pain due to osteoarthritis. A physical examination showed an alert, oriented, and frail elderly lady with ecchymosis on her left arm. There were no signs of heart failure decompensation, including peripheral edema or jugular venous distension. An electrocardiogram (EKG) revealed a complete AV block with a junctional escape of 54 bpm and a corrected QT interval (QTc) of 550 ms (Figure 1A). Her metabolic profile, including serum electrolytes, renal function, liver function, and thyroid function tests, was normal. Transthoracic echocardiogram (TTE) revealed an ejection fraction (EF) of 40-45% with a large area of apical akinesis consistent with Takotsubo syndrome (Video 1). Serum troponin at admission was 0.718 ng/ml and it peaked at 1.835 ng/ml, raising suspicion of an acute coronary syndrome. A coronary angiogram was performed, which revealed mild coronary artery disease with a dominant right coronary artery.

**Video 1:** Echocardiogram, apical four-chambered view demonstrating movement of the basal segments but akinesis within the apex.

Her diagnosis of complete AV block and HFpEF was made 15 months ago, in February 2020, when an EKG revealed junctional escape rhythm at 46 bpm, QTc of 470 ms, and upright T waves (Figure 1B). A TTE at that time revealed an LVEF of 65-70% with mild concentric left ventricular hypertrophy but no regional wall motion abnormalities. Right ventricular systolic pressure (RVSP) was measured and elevated at 55-60 mm Hg, indicative of moderate pulmonary hypertension. Enalapril and Lasix were started, and she was advised to get a permanent pacemaker implanted. The procedure, however, was postponed by the patient and her family due to the COVID-19 pandemic, and she was discharged home for outpatient follow-up.

The patient ended up receiving single-chamber pacemaker implantation this time. Dual-chamber pacemaker was deferred because her skin was too thin, and she did not have enough subcutaneous tissue to place additional leads. Her symptoms improved after the procedure and a TTE repeated after seven days showed marked improvement in LV function with an ejection fraction of 55-60%.

## Discussion

Takotsubo syndrome was first described in 1991 and got its name for the visual similarities between the Takotsubo (octopus pot) and the left ventricular apical ballooning [1]. It is a reversible form of acute heart failure, and the underlying factor is usually overactive sympathetic activation due to emotional or physical stress [2]. Although previously considered a benign condition, it is now estimated that approximately 52% of patients with TTS experience some complications such as acute heart failure, right ventricular involvement, cardiogenic shock, left ventricular thrombus, atrial fibrillation, and CHB [2,3]. A nationwide cohort analysis by Patil found that of 56,000 cases of TTS, 0.7% had coexisting CHB, and the majority of these required permanent pacing [9]. A crucial question about the link between TTS and CHB remains unanswered, as there are several possibilities: CHB is a physiological stressor that triggers TTS or TTS causes CHB, or the link is accidental with no causality. Several case reports can be found in the literature, some documenting CHB as a cause of TTS and others noting vice-versa [4-8]. Our case is unique since we can document the temporal relationship between the rhythm disturbance and the subsequent TTS. The patient was diagnosed with CHB over a year before presenting to the hospital with what initially appeared to be acute coronary syndrome. Eventually, it was discovered that the patient had TTS. The delay between the diagnosis of CHB and the implantation of a permanent pacemaker was due to the COVID-19 pandemic. This is a one-of-a-kind case where CHB was documented and left untreated long enough for TTS to develop. Her medications did not list any iatrogenic causes of CHB, and her transthoracic echocardiogram did not show evidence of any infiltrative processes. There was no indication of leukocytosis or fever to suggest systemic infection, and no significant electrolyte/metabolic disturbances were observed. Based on the history and present illness, the management plan relied on the implantation of a permanent pacemaker and supportive therapy.

## Conclusions

Takotsubo syndrome is infrequently accompanied by a complete heart block. Our case involves an untreated advanced conduction disease that resulted in TTS. The presence of complete heart block prior to the onset of TTS supports the hypothesis that the stress of AV block is the cause rather than the result of TS. In such cases, a permanent pacemaker should be implanted as soon as possible.
